# The Welfare and Educational Impacts of Encounter Experiences and Displays on Zoo‐Housed Red Panda (*Ailurus fulgens*)

**DOI:** 10.1002/zoo.70041

**Published:** 2025-11-21

**Authors:** Sarah L. Spooner, Rebecca N. Lewis, Ewan Davies, Katherine Whitehouse‐Tedd, Leah J. Williams, Kanako Tomisawa, Mark J. Farnworth

**Affiliations:** ^1^ School of Animal Rural and Environmental Sciences, Nottingham Trent University Southwell UK; ^2^ Conservation Science, Chester Zoo, Upton Chester UK; ^3^ School of Biological Sciences, University of Southampton Southampton UK; ^4^ Omuta City Zoo Japan; ^5^ Kyushu University Fukuoka Japan; ^6^ Moulton College Moulton Northamptonshire UK

**Keywords:** ambassador animals, human‐animal interaction (HAI), reproduction, survival

## Abstract

Close animal encounters potentially increase visitor connection to species and present an educational and fundraising opportunity. However, evidence of the impacts on animal welfare or visitor education is limited. Red panda (*Ailurus Fulgens spp*.) encounters are gaining popularity despite a lack of research on their effects. As red panda are a characteristically cautious species and prone to disturbance, concern has been raised as to their suitability for encounters. We examined the extent and composition of red panda encounters amongst 150 Global Species Management Plan (GSMP) member zoos (survey responses), and their impact on longevity and reproduction (species 360 analysis). Over a third (39%) of zoos surveyed offered red panda encounters, with most (71%) being animal feeding experiences. Educational information was provided in almost all cases (95%) and focused on the encounter individuals and species’ natural history. Of the 31 encounter red panda who were also part of a breeding program, 24 reproduced. Comparative data analysis suggested that encounter red panda produced more offspring and had higher longevity (survival) than non‐encounter individuals, although this may reflect changes in red panda husbandry over time. *A. f. styani* were less likely to breed and produced fewer offspring than *A. f. fulgens*. Whilst there appears to be no major negative impacts of red panda encounters, continued monitoring and ensuring high animal‐welfare standards remains vital.

## Graphical abstract

We investigated the extent and composition of red panda (*Ailurus Fulgens spp*.) encounters amongst 150 global zoos and their impact on animal longevity and reproduction. Comparative data analysis suggests that encounter animals produced more offspring and had higher longevity (survival) than non‐encounter animals with differences noted between subspecies.

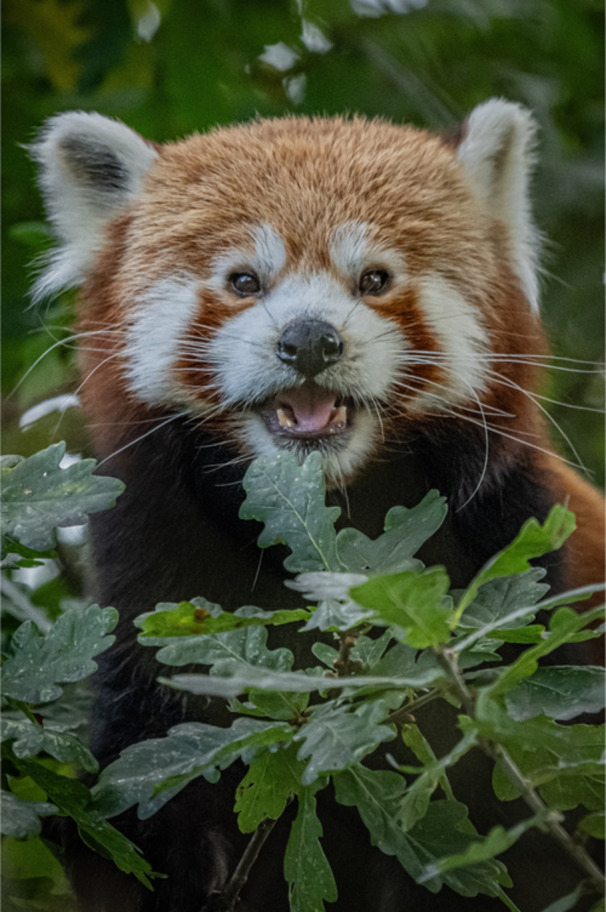

## 1 Introduction

Human‐Animal Interactions (HAIs) are thought to enable visitors to make connections with species and inspire visitors to support conservation efforts (Clayton et al. 2017; Fuhrman and Ladewig 2020; Luebke et al. 2016; Skibins and Powell 2013). These encounters generate revenue for conservation (Ward and Sherwen 2018) and are a potential educational opportunity (Baird 2018; de Mori et al. 2019; Farmerie 2018). Globally, over 75% of zoos offer animal encounters involving various taxa (D'Cruze et al. 2019). These include direct or indirect interactions (D'Cruze et al. 2019; Spooner et al. 2021; Whitehouse‐Tedd et al. 2018).

However, there are concerns that close‐contact encounters with trained wild animals could create false assumptions of tameness and lead to visitors desiring them as exotic pets (Nekaris et al. 2013; Ross et al. 2011). Further concern is raised over whether hand‐rearing or training could increase abnormal or stereotypical behaviors (D'Cruze et al. 2019) or increase stress responses (Lynn 2008). In contrast, other studies suggest encounters have no impact on behavior (Baird et al. 2016) and may even benefit animal welfare by acting as enrichment (Acaralp‐Rehnberg et al. 2020; Sherwen and Hemsworth 2019). Spooner et al. (Spooner et al. 2021) conducted a systematic literature review to investigate the known welfare impacts of close‐contact visitor encounters on zoo encounter individuals. This revealed mixed impacts dependent on species with some concern over the long‐term implications.

Encounter experiences are considered, in this context, as situations where visitors are given close‐contact access to a species. Examples include visitors being inside the animal's enclosure, feeding or touching the animal, or taking close‐proximity photographs with the animal. Simply viewing the animal within its enclosure is not considered an encounter. In addition to encounters, animals may take part in educational displays. These include keeper talks (where the animal remains within its enclosure, exhibiting natural behaviors), animal training (where animals perform on‐command behaviors within their enclosure as part of normal husbandry), and animal shows (where animals perform on‐command behaviors for the benefit of the public, often in a separate area to their normal enclosure). Depending on how intrusive, or how much the experience deviates from natural behaviors, the type of encounter or display could impact animal welfare.

The red panda *(Ailurus fulgens)* is an IUCN Red List Endangered Species commonly housed in zoos (Glatston et al. 2015). The species has recently been classified into two subspecies, *A.f. fulgens* and *A.f. styani*, based on biogeography and morphology, with *A.f. styani* having lower instances of triplets and quadruplets (Curry 2022), and a reduced number of fertile years despite an increased longevity (Curry 2022; Glatston et al. 2022; Hu et al. 2020). The extent of these differences and their impact on husbandry is not yet fully understood (Glatston et al. 2022). According to current [at the time of writing] zoo records (ZIMS) there are 152 *A. f. styani*, and 525 *A. f. fulgens* housed in zoos globally, with a further 114 red panda not classified into subspecies.

Red panda breed well in zoos and in recent years have produced more offspring than expected. This has led to an upwards trend in captive population numbers and concerns over available space. Whilst red panda are typically housed in breeding pairs, many zoos house same sex groups to slow down this population growth (Kappelhof and Weerman 2020; Weerman 2021a).

The red panda International Studbook and Global Species Management Plan (GSMP) have noted an increase in red panda encounters in recent years. Yet there have been no studies to document this trend or investigate impacts (Spooner et al. 2021). Red panda are crepuscular, with activity fluctuation throughout the year depending on temperature, food availability, or season (i.e., breeding) (Kappelhof and Weerman 2020; Weerman 2021b), therefore, they are not naturally active during visitor hours. Further, the species are reported to be reclusive and sensitive to disturbance (Glatston et al. 2022; Kappelhof and Weerman 2020), suggesting they may not be the best species for close‐contact encounters with novel individuals (i.e., visitors).

Due to the lack of empirical data on the effects of these encounters, multiple ethical questions remain, including whether selecting for desirable personalities is unintentionally modifying the captive population, or whether encounters are impacting breeding success or longevity (Glatston et al. 2022; Kappelhof and Weerman 2020; Weerman 2021b). As such the European Association of Zoos and Aquariums (EAZA) Best Practice Guidelines currently recommend that red panda do not participate in visitor encounters until their impact is understood (Weerman 2021b).

With these concerns in mind, this study aims to examine: (1) the frequency and types of red panda encounters offered within GSMP member zoos; (2) the conservation education content of red panda encounters and displays; (3) whether there are any characteristics being selected for in encounter individuals; (4) the current welfare monitoring for encounters; (5) the potential short and long‐term impacts of red panda encounters on animal behavior, longevity, and reproductive success.

## 2 Materials and Methods

### 2.1 Survey Data

Between November 2021 and July 2022 an in‐depth online survey (hosted on the JISC platform [www.onlinesurveys.ac.uk], see supporting materials S1) was designed in conjunction with, and distributed by, the Red Panda Network (RPN) and GSMP coordinators. It was sent to all red panda GSMP member zoos (n = 522). The survey was presented in English and Japanese. English is the standard language of all GSMP documents and it was therefore assumed that the majority of GSMP members could understand surveys written in English. The Japanese translations were produced at the request of the GSMP due to concerns regarding language barriers. We acknowledge that this creates bias towards English and Japanese speaking countries and may have impacted response rates. Chinese zoos are not members of the red panda GSMP and were therefore excluded from the survey, despite many Chinese zoos housing red panda.

The survey consisted of five sections including closed and open‐ended questions. These included: Information on the zoo and individual completing the survey (5 items): Whether red panda encounters took place, including specific details about where, when, and how, whether food/rewards were provided, and/or educational information given (23 Items); Information (global identifiers, behaviors, personality traits (as rated by animal care staff), breeding record) on a specific encounter red panda chosen at random (the individual with the longest name was selected) (5 items); The Global Accession Number (GAN) of all red panda involved (currently or historically); Whether historic encounters had taken place, including dates, details of the animals historically involved and, if relevant, why encounters were stopped (3 items); Whether red panda are involved in educational displays, the nature of these displays and educational information provided (9 items).

The survey took around 45 min and used skip logic, ensuring that only relevant sections of the survey were completed, thus reducing survey fatigue. The survey could be paused and returned to at any point during the 8‐month response period. Reminder emails were sent at midpoint and a month before the survey closed.

Survey responses were examined in Microsoft Excel and descriptive statistics produced.

### 2.2 Species 360 Data

Using Species 360, combined with survey information, we retrieved details from all red panda housed (currently and historically) within the zoos that responded to the survey. We only included red panda born after 1990 (zoo practices before this date differed substantially to current), and that were older than 1 year. Data were extracted with a fixed end date (2023/06/01). Animals where date of birth, sex, or subspecies were unavailable or where information was ‘lost to follow‐up’ were excluded from the data set. As a result, 1076 red panda were included in the final data set.

For each individual, we extracted information on the zoo where the individual was most recently housed, the location of the zoo (country), the subspecies (*A.f. fulgens* or *A.f. styani*), and the identity of the sire and dam. Unknown parents were given a unique identifier in the data set. We also extracted birth and death date (where applicable) and calculated age in days. For living individuals, age at 2023/06/01 was calculated. We extracted information on breeding including whether the individual had bred, the number of offspring, and the number surviving beyond 1 year. Finally, we extracted information on the number of lifetime transfers (to a different zoo) and the age at first transfer for each individual. In some cases, the number of moves was unclear based on the available data and so the move data for these individuals (*n* = 30) was not recorded. Species 360 data were combined with survey information to determine encounter status (has or has not participated in an encounter) for each individual.

Data were analyzed in R (version 4.3.1) (R Core Team 2022). We investigated factors affecting survivorship with a mixed effects Cox regression analysis using the coxme package (Therneau 2020). Most encounter individuals were alive at the time of data collection (111 living individuals out of a total 142 animals), so the use a of Cox regression model, which allows us to consider both living and dead individuals, was a more appropriate method than examining longevity (which can only be calculated for dead individuals). The model included encounter status (whether the individual has ever participated in an encounter), sex, subspecies, parent encounter status (whether either parent had ever participated in an encounter), and the interaction between encounter status and sex as predictors. Parental encounter status was included to determine any cross‐generational effects of encounters. The interaction between encounter status and sex allows for differences in the effect of encounters on male and female red panda. We included the zoo, sire, and dam as random effects. Sire and dam were included as genetic inheritance and early rearing conditions could influence survival, and individuals that share a parent will be more similar than those that do not. The zoo was included as a random effect to account for differences in environmental conditions and management across different zoos.

We investigated the factors influencing whether individuals had bred (n = 602) and, if so, the number of offspring and weaning success, using a series of generalized linear mixed‐effect models (GLMMs) conducted in the glmmTMB package (Brooks et al. 2017). Breeding status (whether an individual had bred) was modelled using a binomial GLMM, as the response variable had only two levels. Offspring number was analyzed using a Poisson GLMM, as the response variable consisted of discrete counts. Weaning success was analyzed using a beta GLMM, as this method is appropriate for modelling proportional response variables (Douma and Weedon 2019). Although beta regression is appropriate for proportional responses, the model cannot handle values of exactly 0 or 1. Therefore, we compressed the data according to x*=(x(n‐1) + 1/2)/2, where x* is the new value, x is the original proportion and n is the sample size (Douma and Weedon 2019).

Models included fixed effects of encounter status, sex, subspecies, parent encounter status, a quadratic effect of logged age in days, and the interaction between encounter status and sex. Zoo, sire, dam, and the crossed effect of sire and dam were included as random effects. Age was included as longer‐lived individuals would have more opportunities to breed, and we would, therefore, expect older red panda to be more likely to have bred or reared offspring than younger individuals. Inspection of the data suggested that the effect of age was not linear, with very old individuals appearing to have reduced reproductive success compared to those of middle age for some factors. To account for this, we included a squared term for logged age in days.

## 3 Results

### 3.1 Collection Information

Questionnaire responses were received from 150 zoos, representing 24 different countries and 29% of the red panda International Stud Book (ISB). Not all zoos completed every question. Where responses were incomplete the data provided was used and sample size adjusted accordingly.

The five countries with the highest response rate were Japan, the USA, Germany, France, and the UK (Table 1). The geographical regions best represented were East Asia, Europe, Oceania, and North America. Underrepresented regions included Africa and the Middle East (only one zoo), and South America (no responses) (Table 1).

**Table 1:** Numbers of zoos represented in the survey, including their global region and percentage of the International Stud Book (ISB) represented.

Country	Global region	No. of Zoos represented	Number of zoos in the region (according to ISB)	No. of zoos who responded to survey	% of ISB represented
China	East Asia	44	17	0	40
Hong Kong			2	0	
India			9	0	
Japan			73	44	
Korea			2	0	
Malaysia			1	0	
Nepal			1	0	
Singapore			2	0	
Thailand			2	0	
Taiwan			1	0	
Austria	Europe	71	5	2	31
Belgium			5	2	
Croatia			1	0	
Czech Republic			10	2	
Denmark			8	1	
Finland			2	0	
France			40	11	
Germany			39	15	
Greece			1	0	
Hungary			7	1	
Italy			8	4	
Latvia			1	0	
Luxembourg			1	1	
Netherlands			15	7	
Norway			1	1	
Poland			10	4	
Portugal			3	0	
Romania			1	1	
Russian Federation			3	0	
Slovenia			1	1	
Slovakia			3	0	
Spain			10	2	
Sweden			5	2	
Switzerland			3	1	
UK & Ireland			49	13	
Armenia	Middle East & Africa	1	1	0	
Türkiye			2	1	10
South Africa			8	0	
Canada	North America	28	11	3	20
USA			131	25	
Argentina	South America	0	1	0	
Mexico			2	0	0
Chile			2	0	
Australia	Oceania	6	17	5	27
New Zealand			5	1	
**TOTAL**		**150**	**522**		**29**

### 3.2 Extent, Distribution and Type of Red Panda Encounters

Of the 150 responses, 39% (59 zoos) offered red panda encounters. Oceania and North America had the highest proportion of zoos offering encounters (67% and 61%), followed by East Asia (45%) and Europe (27%).

**Figure 1:**
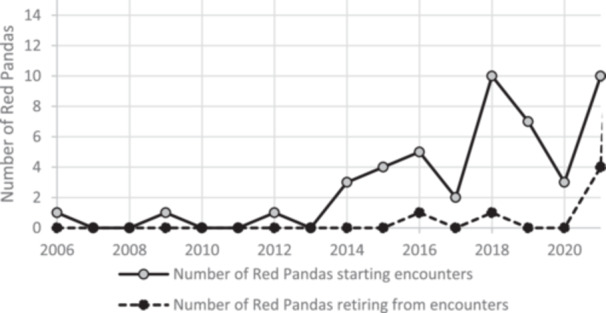
The number of red panda that started participating in encounters annually (solid line) and the number of red panda that retired from encounters annually (dotted line) (*n* = 52).

There appeared to be an upward trend in the number of red panda participating in encounters with a steady increase post‐2013 (Figure 1). Overall, more red panda entered encounter programmes than retired, however there was an increase in retirements around the year 2020, coinciding with the coronavirus pandemic.

The average red panda encounter lasted 20 min (95% CI: 17–22 min, *n* = 55), with on average six people participating (95% CI: 4–8 people, *n* = 56). Animal feeding encounters were the most common (71%), with a fifth of zoos offering multiple types of red panda experience (Table 2). Encounters tended to be daily (30%), in the morning (58%), and coinciding with normal feeding (49%). Where encounter timings were not consistent (32 zoos), six factors determined encounter timings: the behavior (5 zoos) and nutritional needs (3 zoos) of the red panda, the wishes of the visitors (12 zoos), staff needs/availability (5 zoos) and weather conditions (1 zoo). Two zoos stated multiple factors, and 10 zoos did not give a reason. Most encounters (63%) took place within the animal's enclosure, and without a barrier between visitor and animal (94%). Visitors were allowed to feed the animals (‘always’ or ‘depending on circumstances’) in 74% of the cases, whilst touching the animals was restricted and only allowed in 21% of cases (Table 2).

**Table 2:** Composition of red panda encounters amongst GSMP member zoos.

About the encounter	No. of zoos that responded	Details	% of zoos (no. of zoos)
Encounter type	42	Feeding encounters	71 (30)
		Training demonstrations	17 (7)
		Photography	12 (5)
		Behind‐the‐scenes tours	12 (5)
		Multiple encounter types	19 (45)
Frequency	57	Once a day	30 (17)
		Multiple times a day	25 (14)
		Once weekly	28 (16)
		Monthly or less frequent	18 (10)
Timings	57	Same time and place every day	44 (25)
		Morning encounter (08:30 – 11:30)	58 (33)
		Afternoon encounter (12:00 – 16:00)	31 (18)
		Coincide with regular feeding	49 (28)
Location	56	Inside animals' normal enclosure	63 (35)
	35	Was a barrier used?	—
		– No barrier	94 (33)
		– Barrier	6 (2)
	56	Outside animals’ normal enclosure	37 (21)
	21	Was a barrier used?	—
		– No barrier	38 (8)
		– Barrier	62 (13)
Reinforcement	57	Positive reinforcement used	86 (49)
		– Treats (food reward – mainly apples)	79 (45)
		– Praise (verbal)	9 (5)
		– Enrichment	9 (5)
		Treats also offered outside of encounter period	83 (47)
Feeding the animal	57	No visitor feeding	26 (15)
		Visitor feeding always allowed	25 (14)
		Visitor feeding under certain circumstances	49 (28)
Touching the animal	57	No visitor touching	79 (45)
		Visitor touching always allowed	7 (4)
		Visitor touching under certain circumstances	14 (8)
	12	Depending on animal's behavior	50 (6)
		Only when animal feeding	25 (3)
		At staff discretion	17 (2)
		Circumstances undefined	8 (1)
Participation	55	One animal	42 (23)
		Two animals	49 (27)
		More than two animals (Max. 4)	9 (5)
	56	Always use the same individual animal	50 (28)
		Different animals used	—
	35	Used a schedule (rotation)	17 (6)
		Based on health checks	15 (5)
		Based on animal's personality (as rated by animal care staff)	27 (9)
		Animals specifically trained	4 (1)
Staffing	37	Led by a single staff member	59 (22)
		Led by 2 staff members	34 (13)

Most encounters were led by one staff member (Table 2), most likely to be a keeper (19 zoos said ‘always present’) or combined keeper/trainer/presenter (31 zoos said ‘always present’). In contrast, veterinary and research staff were generally absent (4 zoos ‘sometimes’ had veterinary staff and 2 zoos ‘sometimes’ had research staff present).

Around half of the zoos (52%) stated that they conducted formal welfare assessments of their encounters (Table 3) with 18% using an ethogram to collect data. All respondents described some form of informal observational/behavioral monitoring as a means of deciding whether an animal should participate in an encounter. These included ensuring the animal expressed ‘normal’ behavior (23%), although this state was not defined. Other monitoring included observing for increased respiration rate (8 zoos), pacing (6 zoos), and aggression behaviors (2 zoos). When asked how an animal was selected for an encounter 38 zoos said that all animals have the choice to participate, 15 zoos stated they selected based on animal's personality (as rated by the animal care staff) and 2 zoos stated that all animals were trained to participate.

**Table 3:** Reported welfare and behaviors monitoring of encounter red panda.

About the encounter	No. of zoos that responded	Details	% of zoos (n = number of zoos)
Welfare	56	Formal welfare assessment conducted	52 (29)
		– Used ethograms	18 (10)
		– Did not use ethograms	34 (19)
Behavior	56	Monitored behavior	100 (56)
		Specific behaviors monitored:	
		– Eagerness to participate	25 (14)
		– Expresses normal (undefined) behavior	23 (13)
		– Respiration rate (not panting)	14 (8)
		– Eating/good appetite	13 (7)
		– Not pacing (repetitive locomotion)	11 (6)
		– Body condition	9 (5)
		– Not huffing/barking (aggression)	4 (2)
		– Body language/eye expression	4 (2)
		– Animal performs expected behavior (response to a bell/stand at station)	4 (2)
		– Outside temperature	2 (1)

### 3.3 Individual Encounter Animal Information

**Figure 2:**
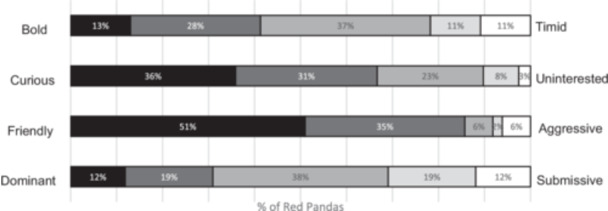
Personality traits (as rated by animal care staff) of the red panda which participate in encounters (*n* = 54).

Encounter red panda was noted as having a friendly and curious personality according to rankings provided by animal care staff (Figure 2). Dominance traits and shyness (bold–timid) were relatively evenly distributed across encounter red panda.

Detailed information was provided for 54 encounter red panda, of these, 17 individuals (31%) expressed at least one behavior of concern “always” ( > 80% of the time; 3 individuals) and/or ‘often’ (51–79% of the time; 16 individuals). These included excessive sleeping (3 individuals) taking apparently purposeless, repetitive routes (7 individuals), repeated motion in a localized area (1 individual), excessive grooming (3 individuals), and scent marking at repetitive locations with no investigation (12 individuals). These refer to the red panda behavior throughout the day and not specifically in response to an encounter. As we do not have a comparison with non‐encounter red panda, we cannot conclude whether this is associated with encounters or is a species tendency. This therefore, warrants further investigation.

Out of the 54 encounter red panda, 31 individuals were part of an international breeding program. Of these, 24 individuals bred (77%), producing 17 litters surviving beyond a year (survival rate of 70%). This matches the 2021 EEP red panda cub survival rate (also 70%) for the general captive population (encounter and non‐encounter individuals) (Weerman 2021a).

### 3.4 Educational Value of Encounters and Displays

Of the 55 zoos that responded to questions on educational resources, most (96%) stated that they included an educational talk. Educational content mainly concerned the individual encounter animals (58%), natural history (44%), wild habitat (47%) and conservation status (42%). When asked specifically if conservation information was given 89% said it was. This consisted of information about threats facing species (51%), zoo‐led conservation strategies (31%), how visitors can help (e.g., donations) (13%), and about the RPN (38%). Three zoos specifically mentioned informing visitors about the illegal wildlife trade for exotic pets. Take away resources/gifts were given in 87% of encounters (48 zoos), these included photos (32 zoos), paintings (10 zoos), and certificates (10 zoos). Six zoos provided take‐home information leaflets about red panda.

Educational displays were more prevalent than encounter experiences, with 99 out of 150 zoos offering them. Displays lasted ~20 min (95% CI 14 min–32 min) and occurred daily in 54% of the cases, with 27 zoos only offering them on special occasions such as International Red Panda Day. Educational displays consisted of talk and animal feed (48%), training display with on command behavior (e.g., standing at a station for health checks) (15%), talk only (17%), interactive workshops (7%), and one zoo offered an education display which allowed visitors to feed red panda using fruit on sticks held over a barrier. Keepers/trainers were in the enclosure with the animal in 74% of displays and touched the animals in 50% of displays. Conservation information was reported to be given in 87 zoos, this consisted of: threats facing species (51%), zoo‐led conservation strategies (39%), how visitors can help (14%), and about the RPN (24%). Take‐home resources were provided by 13 zoos, however, four of these zoos stated these were only available on special occasions (e.g., International Red Panda Day). These were mainly souvenir items: photographs (4 zoos) coloring sheets (2 zoos) and badges (2 zoos), however, seven zoos stated they also provided information booklets about red panda.

### 3.5 Historic Encounters and Non‐Encounter Zoos

There were 67 zoos that had never offered red panda encounters. Of these, 39% (26 zoos) stated that red panda encounters “conflict with their zoo's policy for animal‐visitor interactions”, 37% (25 zoos) wanted to minimize human‐animal interactions, 25% (17 zoos) stated that the red panda enclosure was unsuitable, and 22% (15 zoos) stated ethical concerns. These concerns included not wanting to encourage the idea of red panda as pets, beliefs that close encounters did not add educational value or were demeaning, and concerns that red panda were not suitable encounter individuals. However, five zoos stated that they planned to start encounters once their red panda were trained (1 zoo), they had enough staff (2 zoos), and when enclosures had suitable visitor access (2 zoos).

Eight zoos stated that they had previously had red panda encounters but had discontinued them. The reasons for stopping included the coronavirus pandemic (3 zoos), breeding recommendations (1 zoo), animal's age (too old) (1 zoo), and animal's health (1 zoo). One zoo stated they had stopped encounters as visitors appeared only interested in feeding an animal and not in learning about them.

### 3.6 Species 360 Data

#### 3.6.1 Transfer Data

Transfer data were available for 1046 individuals in the data set. Most individuals (*n* = 572; 55%) had moved zoo once. A further 86 individuals (8%) had never moved zoo and 251 (24%) had moved zoo twice. Only 137 individuals had moved more than 3 times (13%, range 3–6).

Among individuals that had at least one transfer (*n* = 960), the median age at first move among individuals was 1.14 years (interquartile range 0.87–1.68 years), and 83% of individuals moved before the age of 2.

#### 3.6.2 Survivorship

**Figure 3:**
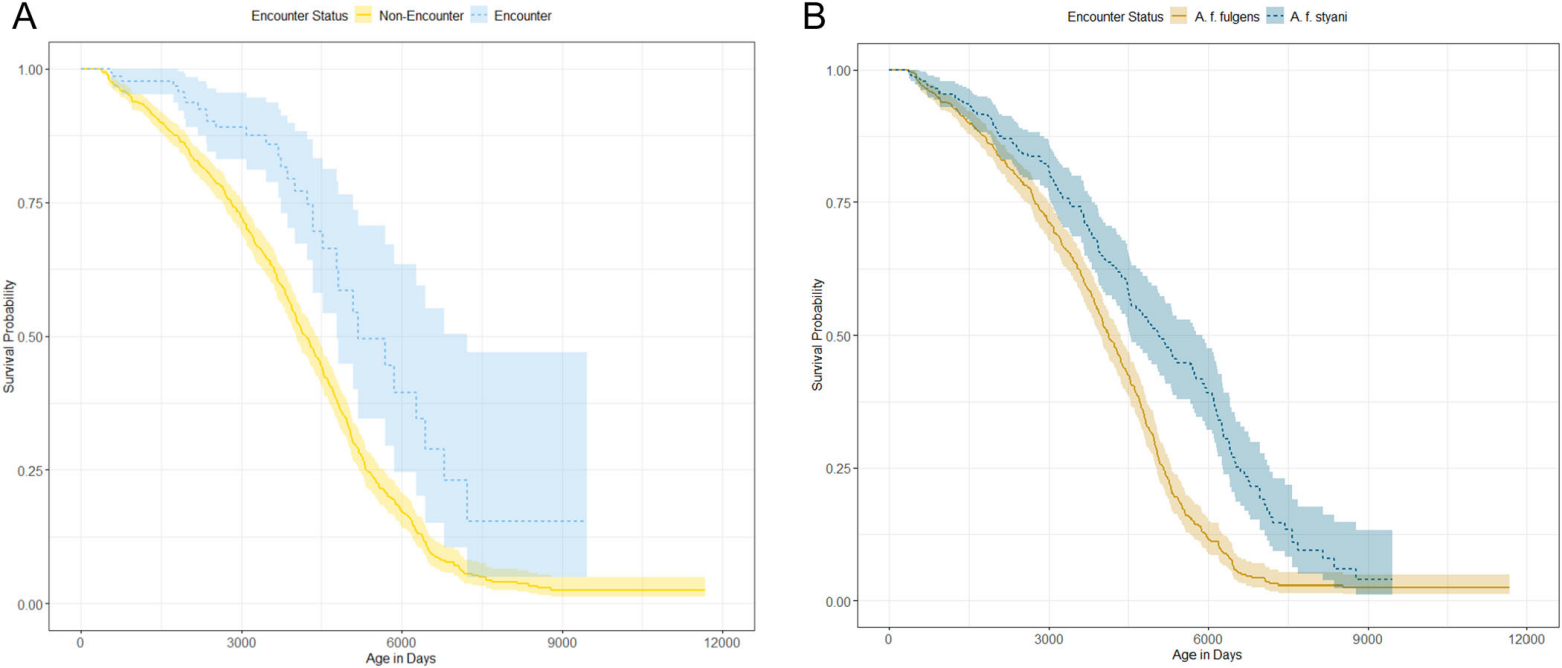
Survival curves showing survival probabilities for a) encounter versus non‐encounter red panda and b) red panda subspecies *A.f. fulgens* and *A.f. styani*.

Individuals participating in encounters had a lower hazard of death (higher survivorship) than non‐encounter individuals (hazard ratio = 0.407, *p* = 0.004). Subspecies also affected survivorship, with *A.f. styani* having a lower hazard of death than *A.f. fulgens* (hazard ratio = 0.526, *p* < 0.001) (Figure 3). Survivorship was not affected by sex (*p* = 0.65), parent encounter status (*p* = 0.69), or the interaction between encounter status and sex (*p* = 0.48).

#### 3.6.3 Reproductive Success

There was no significant difference between encounter and non‐encounter individuals in terms of breeding status (*p* = 0.364, Table 4). However, breeding status was affected by subspecies, with *A.f. styani* less likely to have bred than *A.f. fulgens* (β = −0.826, *p* < 0.001, Table 4) There was no significant effect of sex (*p* = 0.258) or having (at least 1) encounter animal parent (*p* = 0.974), nor was there a significant interaction between encounter status and sex (*p* = 0.456). There was evidence for a nonlinear effect of age, with the likelihood of breeding increasing with age in younger red panda, but decreasing with age in older individuals (Table 4).

**Table 4:** Results of generalized linear mixed effect models examining reproductive success in red panda. Effect sizes and p‐values are shown.

	Breeding status	Offspringnumber	Proportion of offspring weaned
Encounter Status (Encounter)	0.207 *p* = 0.570	**0.276** **p** = **0.011**	*0.475* *p* = *0.053*
Sex (Male)	0.198 *p* = 0.270	**0.104** **p** = **0.024**	−0.030 *p* = 0.792
Subspecies (A. f. styani)	**–0.826** **p** = **< 0.001**	**−0.290** **p** = **0.001**	0.269 *p* = 0.072
Log (Age in Days)	**47.349** **p** < **0.001**	**6.851** **p** < **0.001**	1.607 *p* = 0.108
Log (Age in Days) ^2	**–20.062** **p** < **0.001**	**–1.470** **p** = **0.024**	0.006 *p* = 0.995
Parent Encounter Status (Encounter)	−0.009 *p* = 0.974	0.024 *p* = 0.811	–1.090 *p* = 0.276
Encounter Status (Encounter) * Sex (Male)	−0.361 *p* = 0.456	–0.248 *p* = 0.074	0.334 *p* = 0.738

*Note:* Bold values are statistically significant.

**Figure 4:**
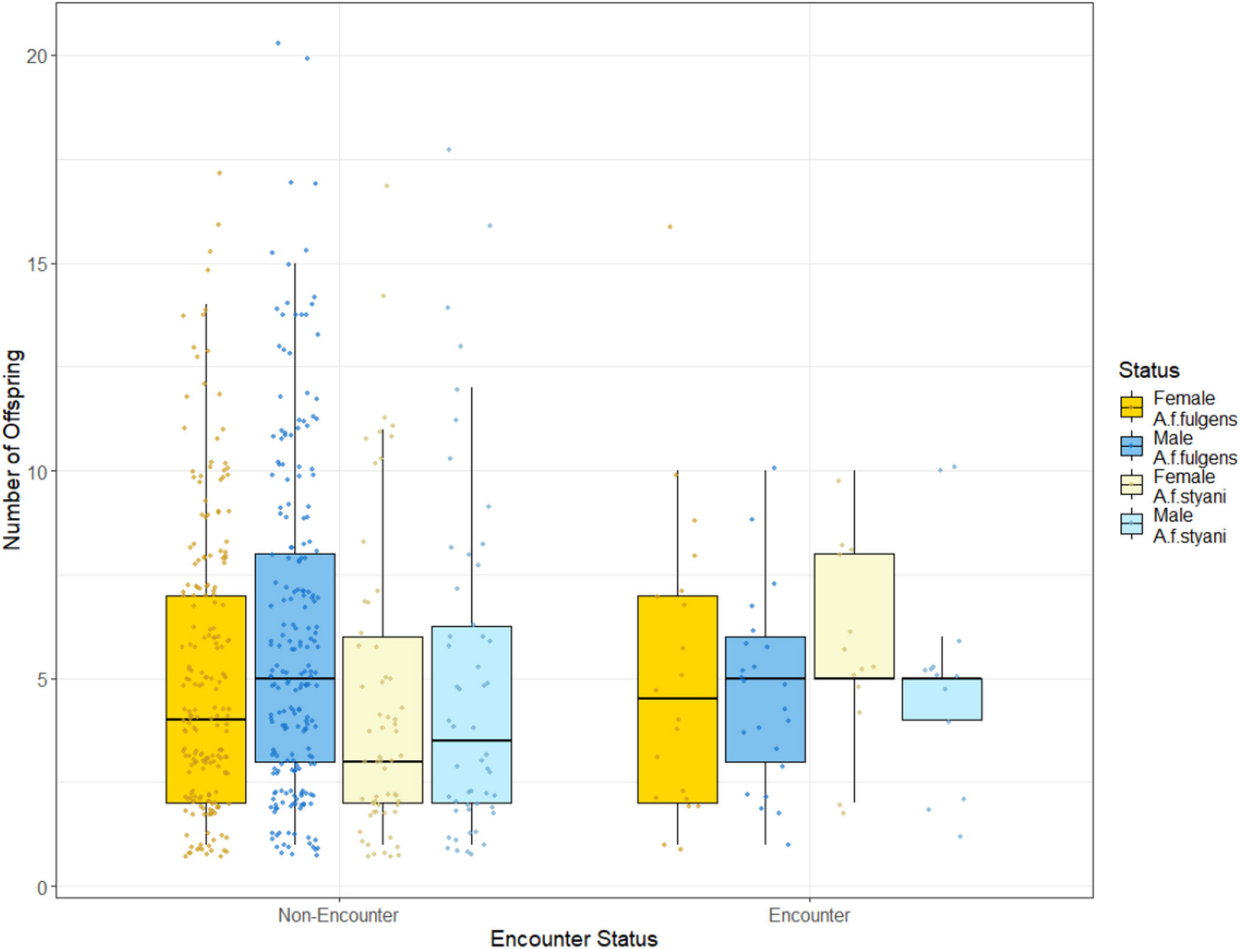
Number of offspring of encounter and non‐encounter red panda among sexes and subspecies. Central lines indicate the median value, with the upper and lower box limits showing the 75th and 25th percentile, respectively.

Encounter red panda had more offspring than non‐encounter red panda (β = 0.276, *p* = 0.011) (Figure 4). Males had more offspring than females amongst non‐encounter individuals (β = 0.105, *p* = 0.024), and there was some evidence for a negative interaction between encounter status and sex, with the difference in number of offspring between sexes reduced among encounter individuals, although this was not statistically significant (β = –0.248, *p* = 0.074). There was a significant effect of subspecies, with *A.f. styani* producing fewer offspring than *A.f. fulgens* (β = −0.290, *p* = 0.001; Figure 4). There was no association between parental encounter status and the number of offspring (*p* = 0.811). There was evidence for a nonlinear effect of age, with the number of offspring increasing with age in younger red panda but decreasing with age in older individuals (Table 4).

**Figure 5:**
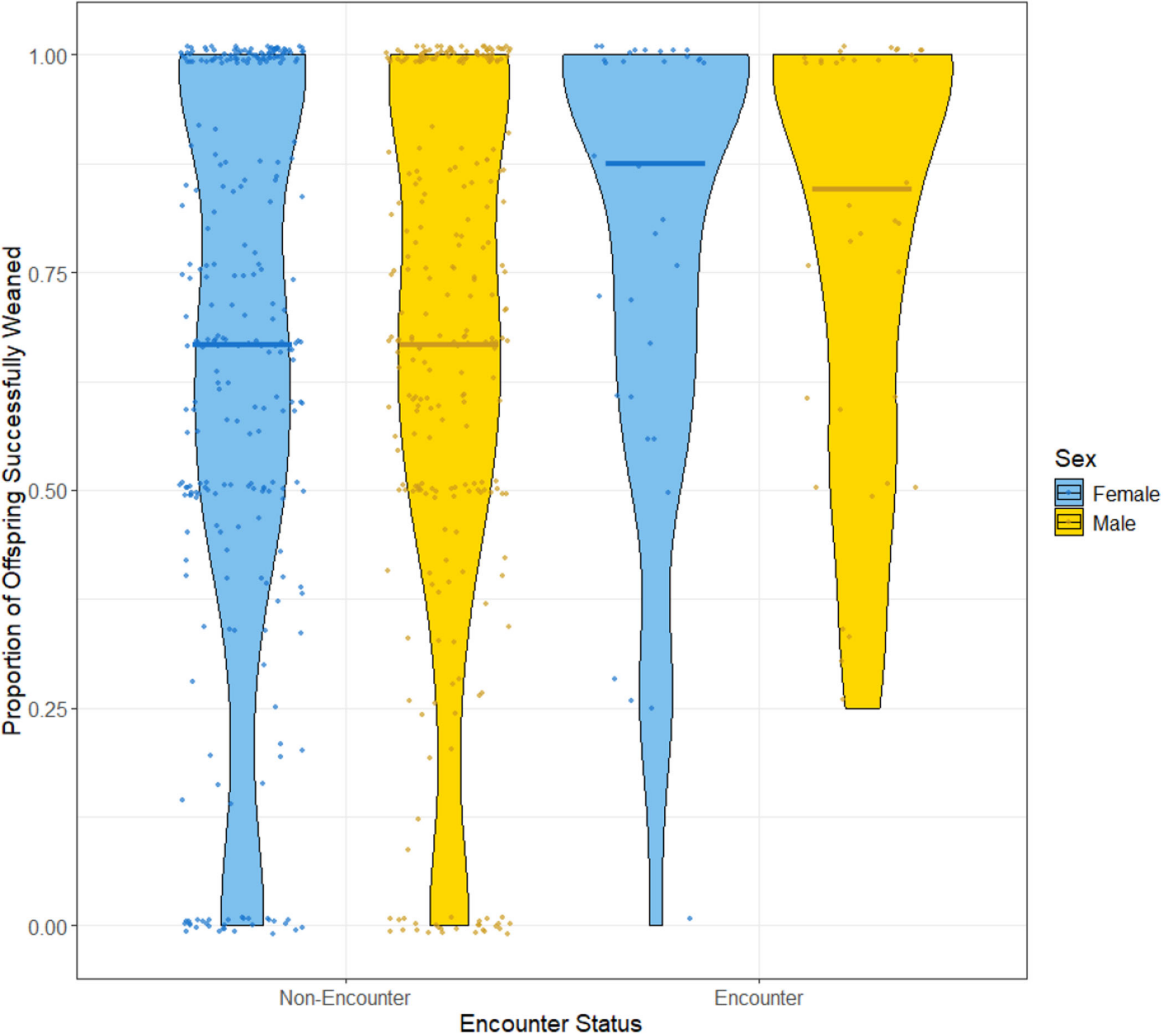
Proportion of offspring successfully weaned (survived to 1 year of age) Solid lines indicate the median value for each group.

The effect of encounter status on weaning success was not statistically significant, although there was some evidence that weaning success was higher among encounter individuals (β = 0.474, *p* = 0.053; Figure 5). There was no significant effect of age, sex, subspecies, parent encounter status, or the interaction between encounter status and sex (Table 4).

## 4 Discussion

The number of red panda encounters offered by GSMP member zoos are increasing and are particularly prevalent in North America and Oceania. Despite concerns that this rise in encounters could negatively affect red panda welfare, we found limited negative effects on behavior and no evidence for a negative impact on breeding and offspring survival within the GSMP.

Despite red panda being viewed as a more reclusive species (Glatston et al. 2022), the red panda involved in encounters were described as having friendly and curious personalities. This suggests potentially personality‐based selection. Personality traits such as increased friendliness have also been identified amongst encounter cheetah (Baird 2018). With increasing numbers of red panda encounters, animal personality may shift with increased time in captivity. This should be monitored as evidence from domestic species suggests selecting for certain personalities can impact species traits long‐term (Careau et al. 2010). This may be a concern if the ex‐situ red panda population becomes behaviorally distinct from that of wild red panda as it may limit future reintroduction opportunities. Whilst there are plans to reintroduce red panda (ZAA, 2024), the vast majority of captive animals will remain in zoos. In these cases, promoting behaviors which favor long‐term captivity may not necessarily be detrimental. It may be that two groups develop, one which retains ‘wild‐type’ characteristics, and one which promotes behaviors suited to long‐term captivity. To an extent this type of selection may already be happening. The GSMP and Red Panda Studbook match pairs of red panda for the purpose of reproduction based on genetics and degrees of relatedness (using PMx software). As such, there are individual red panda who, despite being in excellent health, are not selected for breeding.

Potential behaviors of concern were reported in a minority (31%) of encounter red panda. These were based on reported general observations and not specific tracking pre‐ post‐ and during the encounter. As such, we do not know whether these behaviors are as a result of the encounter or due to other factors. Even when stereotypies occur around an encounter, these can sometimes be explained as anticipatory behaviors and are not necessarily negative. Anticipation behaviors have been noted in several encounter studies (Acaralp‐Rehnberg et al. 2020; Baird et al. 2016; Lynn 2008) and may indicate positive excitement towards the encounter (Szokalski et al. 2013). Animal behavior data were not reported to be regularly collected as part of red panda encounters; therefore, instances of these particular behaviors may be underestimated. Formal welfare assessments during close‐contact experiences are now recommended by a number of zoo associations (e.g. BIAZA Close Contact Policy (BIAZA 2025)). As the study was conducted before this period, it may explain why only half of the zoos surveyed conducted formal welfare assessments during encounters. It is hoped that should the survey be repeated following these updated guidelines, more organizations would adopt behavioral monitoring as standard. Standardized monitoring of encounter individuals through formal welfare assessment is vital to maintain good welfare. Additional behavioral data collection during encounters would provide much needed individual‐level information on the response of each panda to an encounter. As most of the encounters only had one staff member present, who was responsible for both animals and visitors, it would be difficult for one person alone to fully monitor animal welfare whilst also managing the visitor experience. Monitoring several animal‐based (e.g. behavior, physiology) welfare indicators during red panda encounters, over multiple exposures, and at different time points would enable a full picture of encounter impact on red panda welfare to be established.

Red panda involved in encounters had comparatively greater survival and higher reproductive success. Although encounter individuals were not more likely to have bred, they produced more offspring than those not involved in encounters. Although not statistically significant, there was also limited evidence to suggest that encounter individuals were more successful in weaning offspring than non‐encounter individuals.

With the level of data provided by zoos, it was not possible to determine the time periods during which individuals participated in encounters and, therefore, how encounter status relates to an individual's timing of reproductive success i.e., whether offspring were born whilst animals were actively participating in encounters. Whilst this is a limitation to understanding the effect of encounter status on individual's reproductive patterns, our results are still meaningful in the context of the wider GSMP. Our results suggest that current management of encounters is sufficient that reproduction within the GSMP is not compromised by encounter participation; red panda that participated in encounters did not contribute less to the global population over their lifetime than those that did not participate. There is more than one situation in which this could occur, for example, individuals participate in encounters when they do not have a breeding recommendation, individuals outside of reproductive age participate in encounters, or individuals in breeding situations are not impacted by encounters. In reality, these different situations are likely to interact and occur in tandem.

The majority of individuals moved only once across their lifetime, and this usually occurred early in life ( < 2 years old). As a result, the responding zoo is likely to be the zoo where the individual has spent most of its adult life, so reports of encounter status are likely to be accurate. Encounter status is more difficult to determine for individuals with a high number of inter‐zoo transfers, although this affected only a small proportion of individuals ( ~ 13% with 3 or more transfers) in the data set. Removal of these individuals did not alter our findings on the impact of encounter status. Therefore, we expect that the influence of potential misclassification of individuals on our overall results is limited.

We found distinct differences between *A.f. styani* and *A.f. fulgens*, with *A.f. styani* producing fewer offspring. This reflects the findings of Glatston et al. (Glatston et al. 2022) who found that despite having a longer average lifespan *A.f. styani* have a smaller reproductive window (females between 2 and 7 years and males between 3 and 9 years). The *A.f. styani* included in this study were predominantly located in Japan and North America, and it is possible that husbandry practices and environmental factors may also play a role. Our findings indicate that the differences between the two subspecies are still not fully understood and that more work is required in this area.

Red panda are described as prone to disturbance and with high rates of infanticide (Curry 2022; Glatston et al. 2022; Weerman 2021b). However, overall, our results suggest that participating in encounters does not negatively impact reproductive success. Encounters may possibly de‐sensitize animals to human presence. This may be important for ex‐situ populations, however, it could be an issue for in‐situ reintroduction. For example, for Tasmanian devils (*Sarcophilus harrisii*), increased time spent in captivity decreased post‐release survival (Grueber et al. 2017). There may be biases in selection for red panda involved in encounters. Individuals that are less sensitive to novel humans and disturbances may be more likely to be included and may also have greater success in captivity regardless of encounter status. This increased success may also reflect other population‐based changes over time. Alongside the recent increases in encounter popularity, the number of red panda born that go on to participate has also recently increased. There may, therefore, be higher numbers of breeding age animals currently involved in encounter programmes. Improvements in red panda husbandry over time may have contributed to better survival and breeding success in young, compared to old, non‐encounter red panda in the data set (Kappelhof and Weerman 2020). As encounters are becoming more common, only a limited number of animals had encounter animal parents. As a result, multi‐generational patterns are difficult to detect and it would be prudent to continue monitoring multi‐generational effects in the future.

A wide range of red panda encounter experiences were offered by GSMP member zoos, however, animal feeding experiences were most common. This mirrors global encounter preferences (D'Cruze et al. 2019). Most experiences coincided with the animals’ regular feeding times and took place within their enclosure with (on average) six novel visitors. Most zoos stated the animals chose to participate and could retreat if they wished. Whilst we did not find evidence of negative behavioral impacts, some potential ethical concerns were raised. The coinciding of feeding times with encounters could create a conflict between hunger and avoidance, thereby reducing the animals’ control over the situation. The presence of visitors within the individuals’ enclosure may further remove animal choice. A solution may be to provide an encounter area alongside the animals’ regular enclosure where they may choose to visit and interact with visitors (Saiyed et al. 2019). In addition, food should be provided in both areas to ensure that red panda are not forced or coerced into the space because they desire food but are choosing the interaction in itself.

The educational opportunities associated with encounters could be improved. Nearly all encounter experiences included an educational talk with information about the encounter animal, species information, and conservation status. This is in common with the content of other live animal shows and creating some connection to the encounter individuals themselves (Spooner et al. 2021). Efficacy may be impacted because information was delivered at the same time as the experience. We note that one zoo stopped its feeding encounters as they felt visitors were more interested in the experience than in learning, and several zoos did not offer encounters because they felt it did not add anything educational. It is possible that, regardless of educational value, encounters may create emotional connections between visitors and the animal (Howell et al. 2019), however, we did not measure this in this study.

Most encounters and displays included specific conservation content, this was primarily regarding threats facing species and, to a lesser extent, information about zoo‐led conservation strategies, conservation charities (RPN), and actions visitors can take. A common criticism of zoo conservation content is that not enough emphasis is placed on empowering visitors to act (Ballantyne and Packer 2016; Macdonald 2015; Mann et al. 2018) and tends to focus on the negatives (threats) without offering practical solutions (Routman et al. 2022). Some zoos focused on information about why red panda do not make good pets. While this is a threat, it is only relevant in countries where keeping red panda as a pet is allowed. Actions that are irrelevant to visitors’ lives may be simply ignored or could increase a sense of blaming ‘others’ for conservation issues and not taking responsibility themselves (Luna 2023). It is therefore critical that zoos place greater emphasis on relevant conservation actions that visitors can take as well as supporting the zoo's own efforts.

Take home resources have been found to increase educational impacts by increasing visitor awareness offsite (Ballantyne and Packer 2016; Bueddefeld and Van Winkle 2018). Whilst take‐home resources were offered by some zoos for both encounters and displays these were mainly souvenir items. Take‐home information packs were less common and were often associated with specific events (e.g. International Red Panda Day). Such information packs may be a better alternative, with greater learning potential, than simply giving souvenir mementos.

## 5 Conclusions

Despite concerns over the increasing number of red panda encounters and a lack of explicit behavioral studies to examine the impacts on individual welfare, we found no major concerns based on studbook data and the collection survey. Further, we found that lifetime reproductive success and longevity were not compromised by encounter participation. We identified a potential trend of higher reproduction and survival rates amongst encounter individuals. Whilst this is reassuring for zoos offering red panda encounters, we stress that the data is based on reported monitoring and general analysis of global data and does not account for the nuances of zoo husbandry and animal care. We recommend that next step research should include intensive behavioral monitoring comparing encounter and non‐encounter red panda, both during and outside of encounter periods.

The global surveys identified several key areas for improvement. The first was the need for more consistent behavioral monitoring, especially regarding encounter animals. Behavioral monitoring is a key part of welfare assessments and is increasingly recommended in zoo standards. As encounter red panda were often selected based on certain personality traits (rated by animal care staff), examining the impacts of personality on behavior would also be a useful next step in monitoring the impacts of encounters.

Additionally, we identified potential ethical concerns regarding the format of encounters. In many cases, encounters coincide with feeding and involve zoo visitors within the animals’ enclosure. This potentially causes a behavioral conflict for the animal. More monitoring and adaptations to the encounter set‐up to encourage greater opportunity for animal choice are recommended.

A key argument for encounters is their educational and economic potential. Whilst education is a prominent feature of red panda encounters globally, current messaging focuses on individual animal and species facts. This has minimal value and can be improved by focusing on encouraging personal actions to protect the species. Further research is needed to establish whether an encounter creates emotional attachments or distracts from the educational message being delivered.

Encounters should always have a clear purpose and be undertaken with full consideration of the animals’ welfare and the long‐term implications for species. This study represents an overview of global red panda encounters and their potential impacts. The next critical step is to support this through detailed behavioral monitoring and educational assessments.

## Author Contributions

Conceptualization: Katherine Whitehouse‐Tedd. & Mark J. Farnworth. Methodology: Katherine Whitehouse‐Tedd, Mark J. Farnworth, Sarah L. Spooner. Formal analysis: Rebecca N. Lewis, Ewan Davies, Sarah L. Spooner. Investigation: Sarah L. Spooner, Kanako Tomisawa. Translation: Kanako Tomisawa. Data curation: Sarah L. Spooner, Rebecca N. Lewis. Writing – original draft preparation: Sarah L. Spooner, Ewan Davies. Writing – review and editing: Sarah L. Spooner, Rebecca N. Lewis, Leah J. Williams, Katherine Whitehouse‐Tedd, Kanako Tomisawa, Mark J. Farnworth. Visualization: Sarah L. Spooner, Rebecca N. Lewis. supervision, Leah J. Williams, Sarah L. Spooner, Katherine Whitehouse‐Tedd. Project administration: Sarah L. Spooner, Katherine Whitehouse‐Tedd. All authors have read and agreed to the published version of the manuscript.”

## Ethics Statement

This study received ethical approval from the School of Animal, Rural and Environmental Sciences under application number ARE202142.

## Consent

Informed consent was obtained from all subjects involved in the study.

## Conflicts of Interest

The authors declare no conflicts of interest.

## Supporting information

Supplementary_materials_S.1.

## Data Availability

Data are available from the authors.
