# Supplementary material for: The Welfare and Educational Impacts of Encounter Experiences and Displays on Zoo‐Housed Red Panda (Ailurus fulgens)
Source: Zoo Biol. 2025 Nov 21;45(2):163–77. doi: 10.1002/zoo.70041 (PMC13051758; doi:10.1002/zoo.70041)
Supplement: Supplementary file 1 — Supplementary_materials_S.1. [file ZOO-45-163-s001.docx]

**Red Panda Survey Question Template – NB: Survey was administered using the online Jisc platform which allowed for skip logic**

**
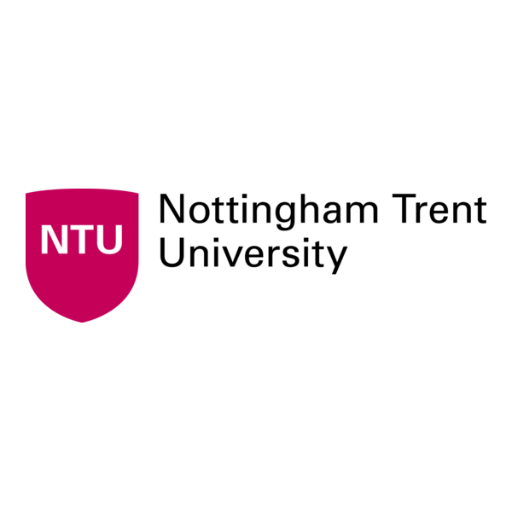
**

**Introductory statement:**

This survey is being conducted on behalf of the Red Panda GSMP to assess the nature of red panda-visitor encounters within global zoo collections. Data collected may be used for other research activities, including subsequent publication. All individual response information will be stored on a password-protected system and will only be accessible to the research team. No personal information will be shared with third parties and all data will be anonymised within the final report. All data shall not be retained for longer than is necessary for the completion of any research projects.

The survey should take around 45 minutes to complete and can be completed in stages (respondents can log back in to continue the survey). We ask that you complete the survey with as much detail as possible.

All participants must be 18 or over. All of the questions are optional and you can choose to stop and withdraw from the study at any time.

If you wish to withdraw you can do so by emailing the project lead, Dr. Spooner, on [sarah.spooner@ntu.ac.uk](mailto:sarah.spooner@ntu.ac.uk) with the name of your organisation. By submitting your data at the end of this questionnaire you assent to their use, unless withdrawn. If you withdraw from the study [by 31st March 2022] any contributions made to the project will be destroyed and your data removed. After this date, data will be anonymised and prepared for analysis. Therefore, they will be unable to be individually identified and cannot be withdrawn. If you have any concerns about the questions or content you should contact Dr. Spooner.

This study has been reviewed for ethical considerations using approved protocols within the School of Animal, Rural and Environmental Sciences and has been approved under application number ARE202142. Any ethical concerns can be raised by contacting [AREEthicalReview@ntu.ac.uk](mailto:AREEthicalReview@ntu.ac.uk). Data will be kept securely in accordance with EU/UK GDPR regulations. Content quality is the responsibility of the academic supervisor who should be contacted at sarah.spooner@ntu.ac.uk with any concerns.

All participating organisations will receive a copy of the completed report sent via email (where an email address is provided) .

We thank you in advance for your participation

I confirm that:

- I am over the age of 18
- I have read the above information and understand the purpose of this study
- I understand that I can stop and withdraw from this study by following the process outlined above
- I understand that the anonymised data I provide will be used for the purposes of research and may be published in writing
- I give permission for the anonymised data I provide to be stored on a password protected file on cloud storage provided by Microsoft so that it may be used for future research purposes
- I understand that any personal information (e-mail addresses, names etc.) that I provide will be destroyed at the end of the project, or prior if so requested.

Confirm (yes/no)

______________________________________________________________________

**Collection Questionnaire:**

**Block 1 Collection Information**

| 1. Name of zoo/collection   [NB: used only for administration purposes, names will be held confidentially and anonymised for reporting]: | free text |
| --- | --- |
| 1. In which region is your collection located? | (drop down)  continent list |
| 1. Is your collection a member of any zoo association? | Yes  No |
| 3a. If yes, please state the names of any organisations that your collection is a member of | free text |
| 1. What is the role of individual/s completing the survey: tick all that apply | (Tick box)   - Curator/ Zoo director - Keeper - Educator/ Presenter - Animal Trainer - Veterinary staff - Other (please specify) |
| 1. Please provide a contact email address [NB: This will be used for administration purposes, including for emailing a copy of the report to you. Names will be held confidentially and anonymised for reporting]: | free text |

**Block 2 Red Panda Encounters**

| Please answer the following about red panda - visitor encounters at your collection:  [When answering please base responses on normal zoo opening and not what has happened during the Covid-19 pandemic] | |
| --- | --- |
| Red panda encounters |  |
| 1. Do you currently offer close-up visitor encounters with your red panda? | Yes  No  (If no – skip logic to block 4) |
| 1. How often do red panda encounters typically (in a non-covid year) take place? | (drop down)   - Several times per day; - Once per day; - Once per week; - Once a month; - Fewer than 10 times per year; - Rarely; |
| 1. What is the average duration of a red panda encounter? | free text |
| 1. Do encounters typically take place at the same time/s every day? | Yes  No |
| 9a. If yes, what times do they take place?  9b. If no, what determines the time of an encounter? | free text |
| 1. How many visitors take part per encounter? If this varies, please provide an estimate of average and the minimum, maximum numbers. | free text |
| 1. Where do RP encounters take place? | (drop down)  - Inside RP enclosure  -Across a barrier (visitors separated from RP)  -in designated area outside RP enclosure  - other |
| 11a. If other please specify | free text |
| during an encounter: |  |
| 1. Do encounters coincide with regular feeding times? | Yes - Always  Yes - Sometimes  No - Never |
| 12a. If yes always: are red panda given access to food other than when participating in the encounter  12b. If yes sometimes: please explain why they only sometimes coincide with feed times | free text |
| 1. Are any reinforcements used during the visitor encounter? (e.g. treats/ praise/ enrichment) | yes  no |
| 13a. if yes, please specify what reinforcements are used | free text |
| 1. If treats/ favourite foods are used in visitor encounters, are these also available during non-encounter periods? | yes  no |
| 1. Are visitors allowed to feed the RP? | Yes - Always  Yes - Sometimes  No - Never |
| 15a. If Yes sometimes - Please explain under what circumstances visitors are allowed to feed the red panda | free text |
| 1. Are visitors allowed to touch the RP? | Yes - Always  Yes - Sometimes  No - Never |
| 16a. If Yes - Please explain when and how visitors are allowed to touch the red panda (please be as detailed as possible) | free text |
| Education |  |
| 1. Is species information given verbally to visitors during the encounter? | yes  no |
| 17a. If yes, please describe the main information given | free text |
| 1. Is conservation information given verbally to visitors during the display? | yes  no |
| 18a. If yes, please describe the main information given | free text |
| 1. Are visitors given anything post encounter? e.g., take home information, photographs etc. | yes  no |
| 19a. If yes please describe, (where possible upload an example) | free text |
| Red panda participation |  |
| 1. How many RP typically participate in each encounter? | free text |
| 1. Please complete the following information for each of the red panda in your collection | \| animal’s GAN \| This animal currently participates in visitor encounter \| This animal currently participates in a display \| This animal has historically participated in an encounter \| This animal has historically participated in a display \| This animal has never participated in an encounter or display \| \| --- \| --- \| --- \| --- \| --- \| --- \| \|  \|  \|  \|  \|  \|  \| \|  \|  \|  \|  \|  \|  \| \|  \|  \|  \|  \|  \|  \| |
| 1. How are RPs selected to take part in an encounter? | (drop down)   - Only those with suitable personality (confident/ calm etc.) selected - All animals trained to participate - All animals have the choice to participate - other reason (please explain your answer) |
| 21a. If other: please explain | free-text |
| 1. Is it always the same individuals that participate in the encounter? | Yes - always  It depends  No |
| 22a. If ‘It depends/ no’: What criteria do you use to select which of your red panda takes-part in each visitor encounter? | free text |
| Staffing of encounters |  |
| 1. How many staff are present during the encounter? If this varies, please provide an estimate of average and the minimum, maximum numbers. | free text |
| 1. What type of staff are present during red panda encounters?(tick all that apply) | \|  \| Always \| Sometimes \| Never \| \| --- \| --- \| --- \| --- \| \| Keeper \|  \|  \|  \| \| Educator/ Presenter \|  \|  \|  \| \| Animal Trainer \|  \|  \|  \| \| Veterinary Staff \|  \|  \|  \| \| Researcher \|  \|  \|  \| \| other \|  \|  \|  \| |
| 25a. If other please specify | free text |
| 1. Is welfare assessed during the encounter? | yes  no |
| 26a. If Yes, please detail how welfare is assessed including stating who assesses welfare during an encounter and which parameters are measured | free text |

**Block 3 Individual animal information**

| The next section will be about one of the encounter red panda in your collection.  We ask you to select one red panda at random. We suggest choosing the animal with the longest name. If there is a tie, we suggest choosing the animal with the most vowels in its name.   - Do not include animals that are under a year old or that are pregnant or feeding cubs. | |
| --- | --- |
| 1. What is the Global Accession Number (GAN) of the selected red panda? | free text |
| 1. Between which years has this red panda participated in encounters? (e.g., between 2016 - 2021) | free text |
| 1. Has this red panda ever reproduced? | no - never attempted breeding  no-attempted breeding but unsuccessful  yes - offspring lived <1 year  yes - offspring lived >1 year |
| 1. Has this red panda ever expressed any of the following behaviours? (please indicate the frequency these behaviours are observed) | \|  \| Has this behaviour ever been seen in any of your pandas? \| Frequency of the behaviour (Always >80% time; Often 51-79% time; On occasion 21-50% of the time; Rarely <20% time; Never) \| \| --- \| --- \| --- \| \| excessive sleeping/ limited response to external stimuli which would normally prompt a reaction e.g., not responding to sudden loud noises \| y/n \|  \| \| Taking a purposeless repetitive route in enclosure/ predictable path (although route can vary to some extent) \| y/n \|  \| \| Purposeless repetitive locomotion (tight circling or moving in a pattern) \| y/n \|  \| \| Scent marking at repetitive locations with no investigation (e.g., sniffing) \| y/n \|  \| \| repeated motion in a localised area facing out towards the public \| y/n \|  \| \| repeated motion in a localised area facing into enclosure \| y/n \|  \| \| Excessive grooming or scratching own body \| y/n \|  \| \| Excessive mouth movements e.g. tongue flicking \| y/n \|  \| \| Excessive grooming of conspecifics (allogrooming) \| y/n \|  \|   Other: please state any other unusual or excessive behaviours that you have noted your RP doing including noting the frequency of these behaviours. |
| 1. please rate the characteristics of this red panda on the following scales: | Dominant 1 2 3 4 5 Submissive  Friendly 1 2 3 4 5 Aggressive  Curious 1 2 3 4 5 Uninterested  Bold 1 2 3 4 5 Timid |

**Block 4 Historic encounters**

| 1. Have you ever (historically) offered red panda public encounters? | No: we have never offered RP encounters  Yes: we have historically but no longer offer them  Yes: we have historically and continue to offer them |
| --- | --- |
| 32a. If no, why have you not offered red panda encounters? | free text |
| 32b. If yes:  If different animals were used to the ones already declared, what were the GAN number(s) of the animal/s used in previous (historic) encounters? | free text |
| 1. Between which years did you offer red panda encounters? | Between (drop down) and (drop down) |
| 1. If you have stopped encounters: what is the reason for stopping RP encounters? | free text |

**Block 4 Education Displays**

| Educational Displays |  |
| --- | --- |
| This section is about educational displays [an ‘educational display’ includes any public talk, demonstration or show where the animal is encouraged to come nearer or be more active in the presence of visitors but do not involve a visitor-RP encounter] | |
| 1. Do your red panda take part in any educational displays? | Yes  No  (if no – skip to next section) |
| 1. During the display does the RP remain inside its normal enclosure? | Yes  No |
| 36a. If no: where is the red panda kept during the display? | free text |
| 1. Is the keeper/handler inside the RP enclosure (or display area if not in its enclosure) during the display? | Yes  No |
| 1. Is the keeper/handler allowed to touch the RP during the display? | Yes  No |
| 1. What is the average duration of each display? | free text |
| 1. What is the frequency of these displays? | (drop down)   - Several times per day - Once per day - Every other day - Once per week - Only on special events e.g. red panda day |
| 1. Is species information given verbally to visitors during the display? | Yes  No |
| 41a. If yes, please describe the main information given | free text |
| 1. Is conservation information given verbally to visitors during the display? | Yes  No |
| 42a. If yes, please describe the main information given | free text |
| 1. Are visitors given anything post encounter? e.g., take home information, photographs etc. | Yes  No |
| 43a. If yes, please describe the main information given | free text |

**Block 5 Future Research**

| 1. Would you be willing to take part in an additional survey (30 minute phone call) about the red panda in your collection? | Yes  No |
| --- | --- |
| 1. Do you have any video footage of your red panda that you would be willing to share with us? We are interested in footage of pandas with and without human (visitor or keeper) presence. | Yes  No |
| 1. Would you be willing to take part in a study to investigate the animal and visitor outcomes of red panda encounters?   [this would involve implementing a welfare evaluation tool which includes behaviour observations, risk assessments, and visitor/staff surveys. It is expected that the commitment would be approximately 4 weeks of work spread across an extended period] | Yes  No |
| 1. Would you like your institution to be listed in the acknowledgements section of any resultant publication? | Yes  No  [Please note that data will remain anonymised within the report] |

**Thank you for your participation.**
